# Kinetic Features of 3′–5′–Exonuclease Activity of Apurinic/Apyrimidinic Endonuclease Apn2 from *Saccharomyces cerevisiae*

**DOI:** 10.3390/ijms232214404

**Published:** 2022-11-19

**Authors:** Aleksandra A. Kuznetsova, Anastasia A. Gavrilova, Alexander A. Ishchenko, Murat Saparbaev, Olga S. Fedorova, Nikita A. Kuznetsov

**Affiliations:** 1 Institute of Chemical Biology and Fundamental Medicine, Siberian Branch of Russian Academy of Sciences, 630090 Novosibirsk, Russia; 2 Group «Mechanisms of DNA Repair and Carcinogenesis», Equipe Labellisée LIGUE 2016, CNRS UMR9019, Université Paris–Saclay, Gustave Roussy Cancer Campus, CEDEX, F-94805 Villejuif, France; 3 Department of Natural Sciences, Novosibirsk State University, 630090 Novosibirsk, Russia

**Keywords:** AP endonuclease, DNA repair, pre–steady–state kinetics, fluorescence, 3′–5′–exonuclease activity

## Abstract

In yeast *Saccharomyces cerevisiae* cells, apurinic/apyrimidinic (AP) sites are primarily repaired by base excision repair. Base excision repair is initiated by one of two AP endonucleases: Apn1 or Apn2. AP endonucleases catalyze hydrolytic cleavage of the phosphodiester backbone on the 5′ side of an AP site, thereby forming a single–strand break containing 3′–OH and 5′–dRP ends. In addition, Apn2 has 3′–phosphodiesterase activity (removing 3′–blocking groups) and 3′ → 5′ exonuclease activity (both much stronger than its AP endonuclease activity). Nonetheless, the role of the 3′–5′–exonuclease activity of Apn2 remains unclear and presumably is involved in the repair of damage containing single–strand breaks. In this work, by separating reaction products in a polyacrylamide gel and by a stopped–flow assay, we performed a kinetic analysis of the interaction of Apn2 with various model DNA substrates containing a 5′ overhang. The results allowed us to propose a mechanism for the cleaving off of nucleotides and to determine the rate of the catalytic stage of the process. It was found that dissociation of a reaction product from the enzyme active site is not a rate–limiting step in the enzymatic reaction. We determined an influence of the nature of the 3′–terminal nucleotide that can be cleaved off on the course of the enzymatic reaction. Finally, it was found that the efficiency of the enzymatic reaction is context–specific.

## 1. Introduction

Maintenance of DNA integrity in cells of living organisms is performed by repair enzymes which detect and remove damaged nucleotides, thus leading to the restoration of the original DNA sequence [1]. One way to remove nonbulky single–nucleotide lesions is base excision repair (BER) [2,3,4,5]. The classic pathway of BER includes sequential action of two types of enzymes: DNA glycosylases and apurinic/apyrimidinic (AP) endonucleases [6,7]. On the other hand, as a result, genotoxic intermediates, such as AP sites and/or blocking 3′–terminal groups, emerge in DNA; they must be removed before DNA repair synthesis is initiated.

The process of DNA damage removal and AP site processing has been studied for many years, and until recently, it has been thought that the main biological function of AP endonucleases is the hydrolysis of the phosphodiester bond on the 5′ side of an AP site, followed by the formation of a 2′–deoxyribophosphate residue at the 5′ end and a 3′–hydroxyl group [8,9,10]. Nonetheless, AP endonucleases can recognize not only AP sites but also some damaged nucleotides containing a modified base and can catalyze phosphodiester bond hydrolysis on the 5′ side of the damaged nucleotide [11]. This DNA glycosylase–independent removal of damaged bases was named nucleotide incision repair (NIR) [12]. Since then, a number of reports have shown that AP endonucleases recognize many structurally diverse types of damage: from bulky benzene adducts of DNA [13] to small oxidatively damaged pyrimidines [14] and other modifications [15,16,17,18,19]. Furthermore, it has been found that some AP endonucleases, aside from participating in BER and NIR, can interact with intact nucleic acids and catalyze 3′ → 5′ exonucleolytic degradation of DNA [20,21,22,23] as well as catalyze cleavage of RNA [24,25,26,27,28].

It should be noted that in mammals, the main AP endonuclease APE1 participates in the processes of BER and NIR [29]. Additionally, a second mammalian AP endonuclease, APE2, has been discovered [30], whose biological functions have not been sufficiently characterized until recently [21,31,32,33]. It has been demonstrated that APE2, in contrast to APE1, exerts strong 3′–5′–exonuclease and 3′–phosphodiesterase actions and has a very weak AP endonuclease activity [21]. In recent years, data appeared in the literature regarding the functional role of human AP endonuclease APE2 as an important participant in DNA single–strand break repair owing to efficient degradation of a DNA strand in the 3′ → 5′ direction [33,34]. 3′–5′–Exonucleolytic hydrolysis of a single–strand break in DNA gives rise to a longer single–strand gap, which in turn activates the general cellular pathway of the DNA damage response. In this context, it was revealed that APE2 is crucial for the removal of 3′–blocking groups at DNA strand breaks that prevent replicative DNA synthesis. Not so long ago [31], a two–stage APE1/APE2–mediated mechanism of DNA degradation at single–strand breaks was proposed that combines the repair of damaged DNA and the repair of single–strand breaks in eukaryotic systems.

In the yeast *Saccharomyces cerevisiae*, two AP endonucleases have been identified too: Apn1 and Apn2 [6]. Apn1 belongs to the Nfo family [35], while Apn2 shares high homology with ExoIII and human enzymes APE1 and APE2 [36]. It should be pointed out that *S. cerevisiae* Apn2 has a higher homology with human protein APE2 than with APE1 [10]. Apn2 has 3′–5′–exonuclease activity with substrate specificity analogous to that of ExoIII [10,37]. Apn2 preferentially hydrolyzes double–stranded DNA substrates and is especially active toward double–stranded DNA having a blunt end and toward a partial DNA duplex having a 3′–truncated end.

In the present work, kinetic features of the 3′–5′–exonuclease activity of Apn2 from *S. cerevisiae* are analyzed and described. The efficiency of hydrolysis of phosphodiester bonds in model DNA substrates was determined by separation of reaction products via gel electrophoresis. By the real–time stopped–flow method, the conformational changes (in both the enzyme and DNA) that are associated with 3′–5′–exonucleolytic degradation of model DNA substrates were recorded for the first time, mathematical processing of the experimental data was carried out, kinetic schemes describing dynamics of the conformational changes were proposed, and catalytic rate constants of the enzymatic reaction were calculated.

## 2. Results and Discussion

### 2.1. Influence of Reaction Conditions on 3′–5′–Exonuclease Activity of Apn2

**Figure 1:**
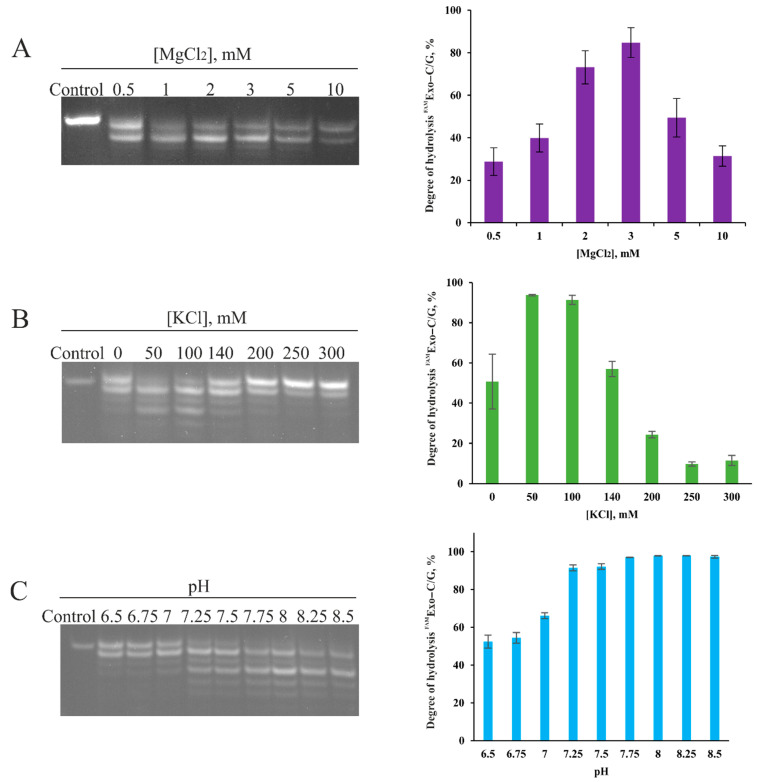
Digestion of substrate ^FAM^Exo–C/G at various concentrations of MgCl_2_ (t = 1 h) (**A**) and of KCl (t = 30 min) (**B**) and at various pH levels (t = 30 min) (**C**). ([Apn2] = 2 μM, [DNA] = 1 μM, T = 37 °C).

Optimal conditions for the 3′–5′–exonuclease reaction of Apn2 were found by means of a model DNA duplex called ^FAM^Exo–C/G. The dependence of the degree of 3′–5′–exonucleolytic hydrolysis of the substrate by Apn2 on the concentration of magnesium and potassium ions as well as on pH of the buffer is presented in Figure 1. As readers can see in the data, optimal concentrations of the ions are: 3 mM [Mg^2+^] and 50 mM [K^+^]. There is also a fairly wide range of optimum pH levels in the buffer: 7.25 to 8.5. The results are in good agreement with the literature data on chimeric protein (GST)–Apn2 [37].

### 2.2. Accumulation Kinetics of Products of the 3′–5′–Exonuclease Reaction

**Figure 2:**
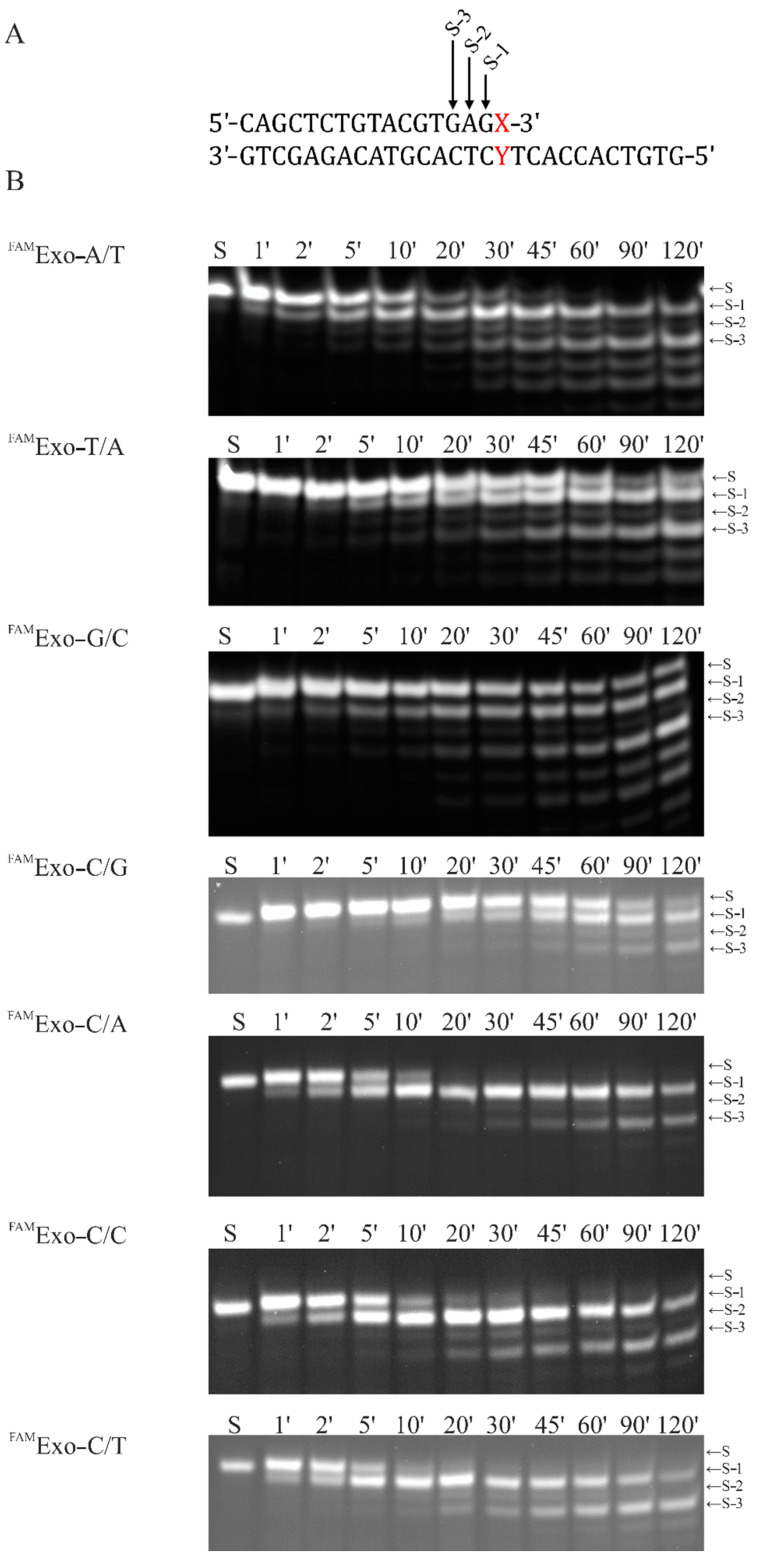
(**A**) An outline of the substrate used. (**B**) Accumulation of 3′–5′–exonuclease reaction products during the interaction of Apn2 with DNA substrates containing a terminal complementary or noncomplementary pair ([DNA] = [Apn2] = 1 µM, T = 37 °C).

To investigate the influence of the nature of the cleaved off 3′–terminal nucleotide on the course of the 3′–5′–exonuclease reaction, substrates with various terminal complementary and noncomplementary pairs were tested (Figure 2A). Kinetics of the accumulation of products of the 3′–5′–exonuclease reaction were characterized by gel electrophoresis (Figure 2B).

It was found that removal of the first 3′–terminal nucleotide and formation of product S–1 ([S–1]/[S] > 50%) took 1 h for fully complementary substrates ^FAM^Exo–C/G, ^FAM^Exo–G/C, and ^FAM^Exo–T/A, but only ~20 min for substrate ^FAM^Exo–A/T, and 10 min for substrates containing noncanonical terminal pairs C/A, C/T, and C/C (Figure 2B). Interestingly, product S–1 was not completely consumed even after 2 h, regardless of the substrate. However, product S–2 corresponding to accumulation of the substrate containing adenine at the 15th position of the hydrolyzable strand (Figure 2A) was found in the reaction mixture only in negligible amounts: it was almost completely hydrolyzed to the 14–mer product.

The obtained data suggest that the 3′–5′–exonuclease reaction proceeded in a context–specific manner. Thus, in the presence of 3′–terminal adenosine, the 3′–5′–exonuclease reaction proceeded much faster as compared to substrates with other complementary terminal nucleotides. Stability of the terminal pair also had a major impact on the course of the 3′–5′–exonuclease reaction. During the digestion of DNA substrates containing a noncomplementary terminal pair, the reaction rate was comparable to that of a substrate containing a 3′–terminal adenine.

**Figure 3:**
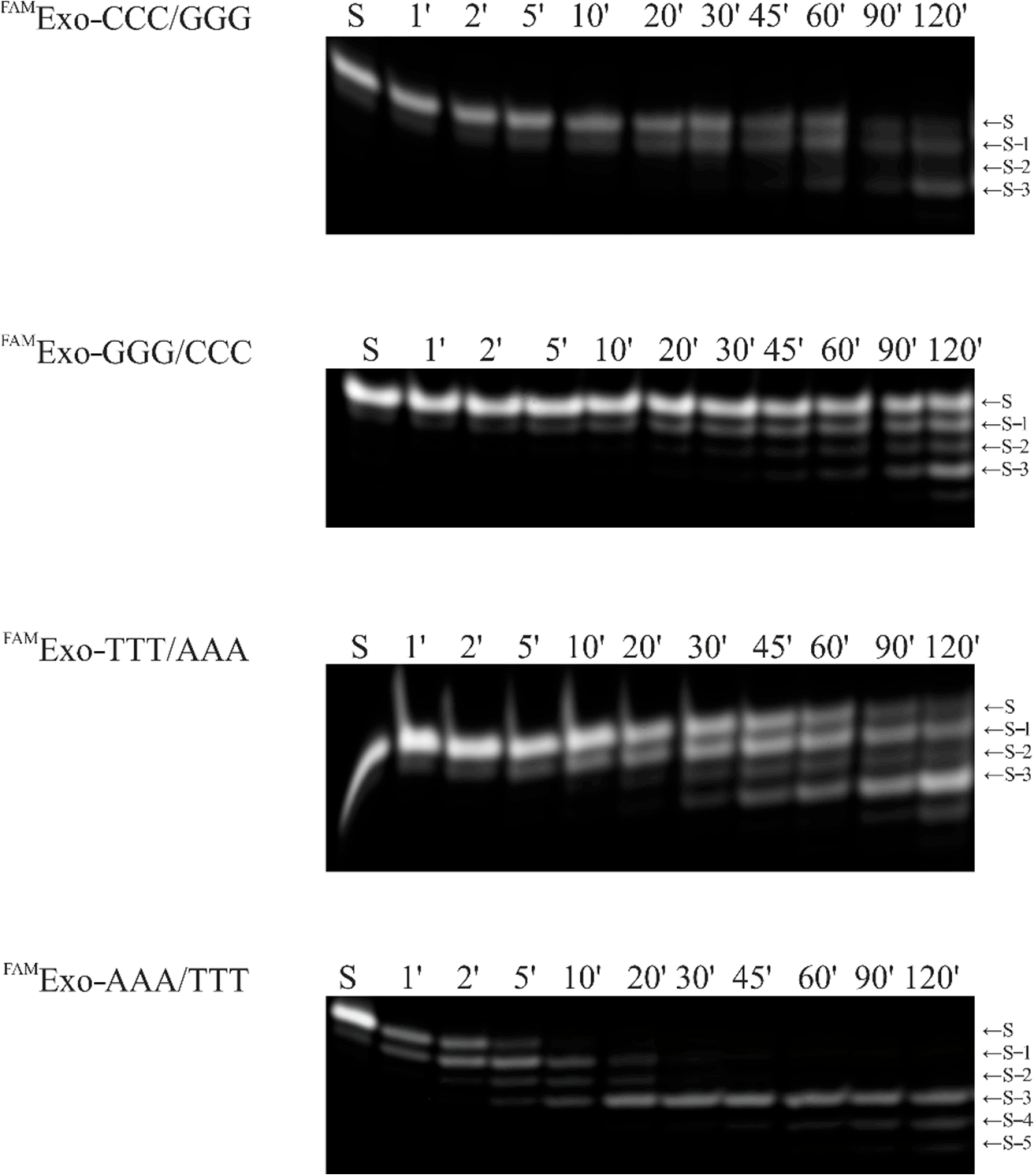
Accumulation of products of the 3′–5′–exonuclease reaction during the interaction of Apn2 with DNA substrates ([DNA] = [Apn2] = 1 μM, T = 37 °C).

To make sure that Apn2 preferentially cleaves off adenosine, a similar experiment was conducted with substrates containing a triad of a nucleotide at the 3′ end (Figure 3). As one can see in the presented data, the presence of 3′–terminal adenine affects the rate of the enzymatic process by substantially accelerating it. The presence of guanosine, thymidine, or cytidine at the 3′ end yielded profiles of hydrolysis of the initial substrate that were similar to one another.

**Figure 4:**
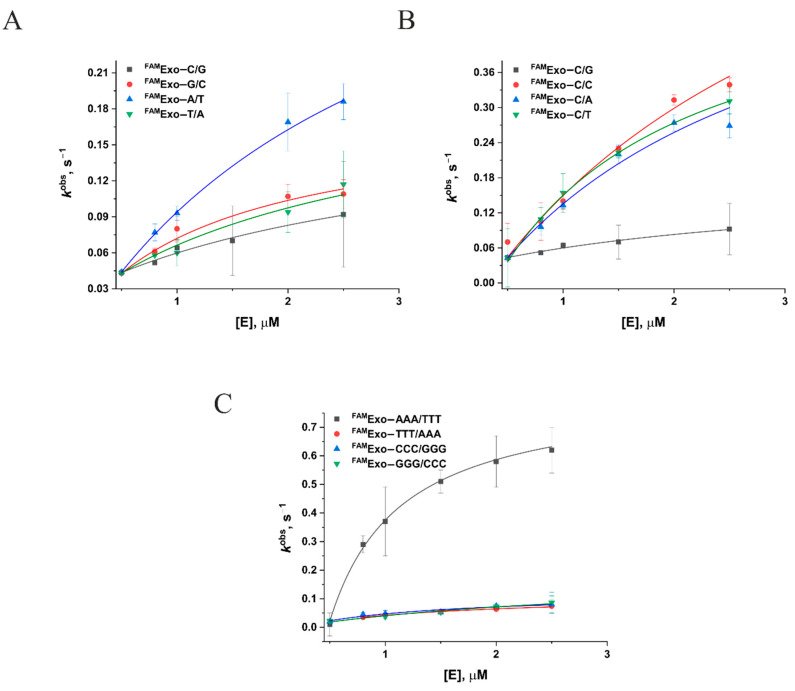
Dependence of the observed rate constant (*k*^obs^) for the formation of 16–mer product S–1 on enzyme concentration for different substrates. (**A**) Fully complementary substrates. (**B**) Substrates containing noncanonical terminal pairs C/A, C/T, and C/C in comparison with C/G. (**C**) Substrates ^FAM^Exo–XXX/YYY with a terminal triad of the same nucleotide. ([DNA] = 1 μM, T = 37 °C).

**Scheme 1:**
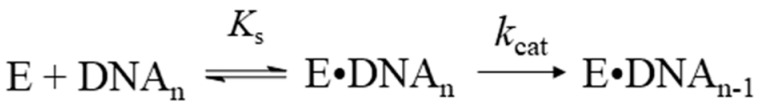
The kinetic scheme of the formation of a complex of Apn2 with a DNA substrate followed by a stage of cleavage of a single nucleotide, where E: enzyme; DNA_n_: a 17–mer DNA duplex; and DNA_n−1_: an S–1 16–mer DNA duplex formed by the removal of the 3′–terminal nucleotide.

The kinetic curves of accumulation and consumption of a product corresponding to the hydrolytic removal of the first nucleotide were fitted to a sum of exponentials; the dependence of the observed rate constant for the formation of product S–1 on the enzyme concentration is given in Figure 4A. It is obvious that the dependence is hyperbolic, indicating a bimolecular process of the formation of the enzyme–substrate complex, which is followed by the catalytic stage (Scheme 1). The dependence of the observed rate constants on the enzyme concentration was fitted to Equation (1), and the resultant values of constants *K*_s_ and *k*_cat_ are listed in Table 1.

(1) $$k^{o}{}^{\mathrm{bs}}=\frac{K_{s}*e_{0}*k_{c}{}_{\mathrm{at}}}{K_{s}*e_{0}+1}$$

where *k*^obs^ is the observed rate constant for the formation of S–1, *e*_0_ denotes initial concentration of Apn2, *K*_s_ represents the binding constant, and *k*_cat_ means the catalytic rate constant of hydrolytic removal of a 3′–terminal nucleotide.

One can see that for the substrate containing the 3′–terminal adenosine (^FAM^Exo–A/T), the catalytic rate constant (*k*_cat_) was approximately two times greater than that for other substrates with a terminal complementary pair and approximately two times less than the catalytic rate constant for substrates with a noncomplementary terminal pair. Constants of formation of the enzyme–substrate complex (*K*_s_) are approximately the same if we take into account the margin of error. This finding suggests that the nature of the terminal nucleotide does not affect the stage of substrate binding. The value of *k*_cat_ for substrate ^FAM^Exo–AAA/TTT significantly exceeds that of all other constants characterizing the catalytic stage of the process. This result confirms our supposition that the presence of 3′–terminal adenine increases the rate of the 3′–5′–exonuclease reaction.

Analysis of the accumulation of products of the 3′–5′–exonuclease reaction during the interaction of Apn2 with DNA substrates (Figure 3) revealed that the amount of product S–2 is still somewhat lower in comparison with S–1 and S–3. Because the obtained data suggest that the efficiency of 3′–end DNA cleavage by Apn2 is dependent on the context of DNA, the rate of relocation of newly formed 3′–end nucleotide of the DNA product within the active site may also depend on the context of DNA. This assumption allows us to explain the variation of the accumulation of different products; however, it requires additional investigation. Indeed, these differences may be due to the architecture of the DNA–binding site, the effect of nonspecific DNA binding by the N–terminal domain of the enzyme, or another reason.

### 2.3. Determining the Inhibitory Action of 2′–Deoxynucleoside Monophosphates on the 3′–5′–Exonuclease Activity of Apn2

During the interaction of the enzyme with a DNA substrate, 3′–terminal nucleotides are removed one by one, the nucleoside monophosphate dissociates from the active site, and the enzyme moves in the 3′ → 5′ direction along the short strand to form a catalytic complex with the next nucleotide. For some endonucleases, for example, APE1 and Nfo, a decrease in enzymatic processivity after the cleavage of the first 3′–terminal nucleotide has been demonstrated [22,23], suggesting that the enzyme is inhibited by the reaction product, i.e., the cleaved off 3′–terminal nucleoside monophosphate can block the active site of the enzyme and prevent the assembly of a new catalytic complex with the shortened product. Furthermore, different nucleoside monophosphates located in the active site of the enzyme can have different inhibitory effects.

**Figure 5:**
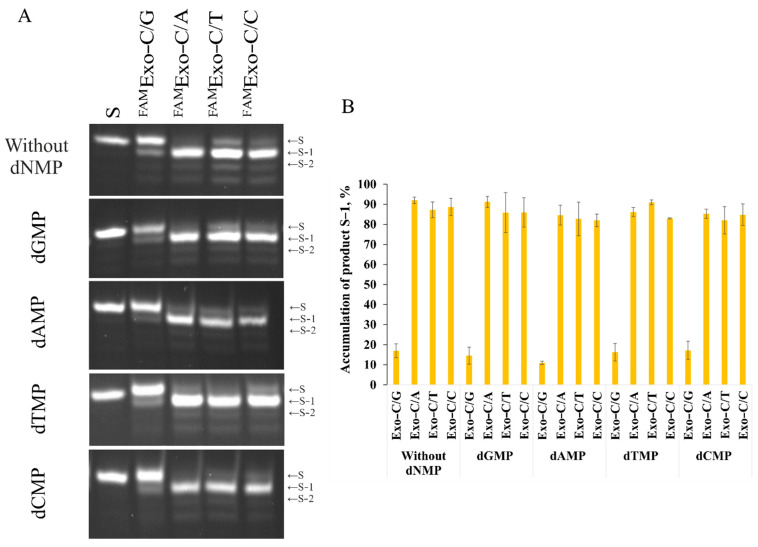
(**A**) Incubation of Apn2 with dNMP ([Apn2] = [S] = 1 μM, [dNMP] = 5 mM, T = 37 °C, t_reaction_ = 10 min). (**B**) The histogram of the accumulation of product S–1 in the presence of dNTP.

To assess the possible inhibitory action of the cleaved off nucleotide, we studied the accumulation of products of digestion of substrates ^FAM^Exo–X/Y (X = C, Y = G/A/T/C) in the presence of various 2′–deoxyribonucleoside monophosphates. For this purpose, the enzyme was incubated for 5 min with a considerable excess of a dNMP (5 mM), after which the enzyme was mixed with a substrate (Figure 5). The obtained data clearly demonstrate that the presence of dNMP in the reaction mixture did not slow down the 3′–5′–exonucleolytic degradation of the substrate (Figure 5B). Consequently, it can be theorized that the release of a dNMP from the active site of the enzyme is not a limiting factor for the 3′–5′–exonuclease reaction. Summarizing the obtained data, it can be concluded that the 3′–5′–exonuclease activity of Apn2 is context–specific and depends on the nature of the cleaved–off nucleotide.

### 2.4. Examination of Conformational Changes of Apn2 during the Enzymatic Cycle

**Figure 6:**
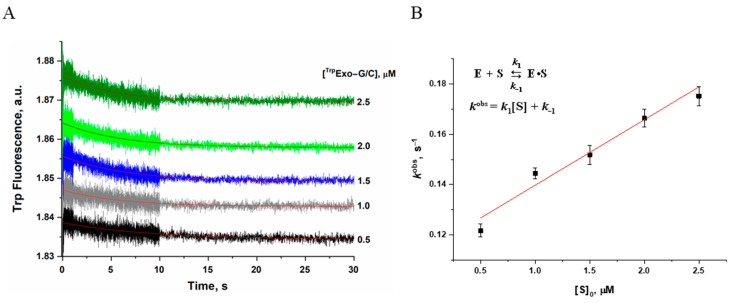
(**A**) The dependence of the fluorescence signal of Trp residues of Apn2 during its interaction with ^Trp^Exo–G/C on the DNA substrate concentration, which was varied from 0.5 to 2.5 μM. (**B**) Dependence of the observed rate constant (*k*^obs^) on the concentration of DNA substrate ^Trp^Exo–G/C ([Apn2] = 2 μM, T = 37 °C).

Apn2 contains five tryptophan residues (Trp70, Trp95, Trp312, Trp498, and Trp519), which could characterize changes of enzyme conformation in the course of interaction with DNA. Changes in fluorescence intensity of Trp residues during the binding and hydrolysis of substrate ^Trp^Exo–G/C are depicted in Figure 6A. At time points < 10 s, a decrease in Trp fluorescence intensity was observed, which most likely denotes the substrate binding and the assembly of the enzyme–substrate complex. The slow reaction of the substrate digestion cannot be registered by this technique because of the destruction of Trp residues during prolonged irradiation at the fluorescence excitation wavelength (the “photobleaching” effect). It is also possible that the environment of Trp residues within Apn2 does not undergo significant rearrangements during the enzymatic cycle owing to specific structural features of this enzyme. 

The initial decrease of Trp fluorescence intensity was fitted to single–exponential Equation (2) to calculate observed rate constant *k*^obs^. The regular residuals of fit curves are presented in Figure S2. The dependence of *k*^obs^ on substrate concentration proved to be linear (Figure 6B), which matched a one–step binding mechanism and enabled us to calculate rate constants for the formation and dissociation of the enzyme–substrate complex (E•S) via Equation (3), yielding *k*_1_ = 0.026 ± 0.004 μM^−1^·s^−1^ and *k*_−1_ = 0.11 ± 0.01 s^−1^. In this case, complex formation constant *K*_1_ is 0.24 ± 0.04 μM^−1^, which is consistent with the results of PAAG electrophoresis.

### 2.5. Analysis of Conformational Changes in the DNA Substrate during the Enzymatic Cycle

Usage of the substrates containing fluorescent analogs of nitrogenous bases such as 2–aminopurine (aPu) or pyrrolocytosine (C^py^) made it possible to register conformational changes in DNA substrates in the course of interaction with the enzyme. It has been shown earlier for human APE1 and Nfo [22,23] that model DNA substrates with a 5′–dangling end containing an aPu residue at different positions from the 3′ end in duplexes Exo–aPu^j^/T (j = 1, 2, 4, or 6) are suitable for 3′–5′ exonuclease activity. A comparison of kinetic data obtained by fluorescence and PAAG electrophoretic analyses for human APE1 and 5′–^32^P–labeled substrates Exo–aPu^j^/T revealed that the phase of the increase in aPu fluorescence intensity coincides with the removal of the aPu nucleotide from the jth position of the hydrolyzed oligonucleotide [22], suggesting that the modified bases do not have an effect on the reaction. To characterize conformational dynamics of a DNA substrate during the catalytic cycle, duplexes were designed containing an aPu or C^py^ residue either at the first and second positions from the 3′ end of the hydrolyzable strand or in the complementary strand opposite the first nucleotide that can be cleaved off.

**Figure 7:**
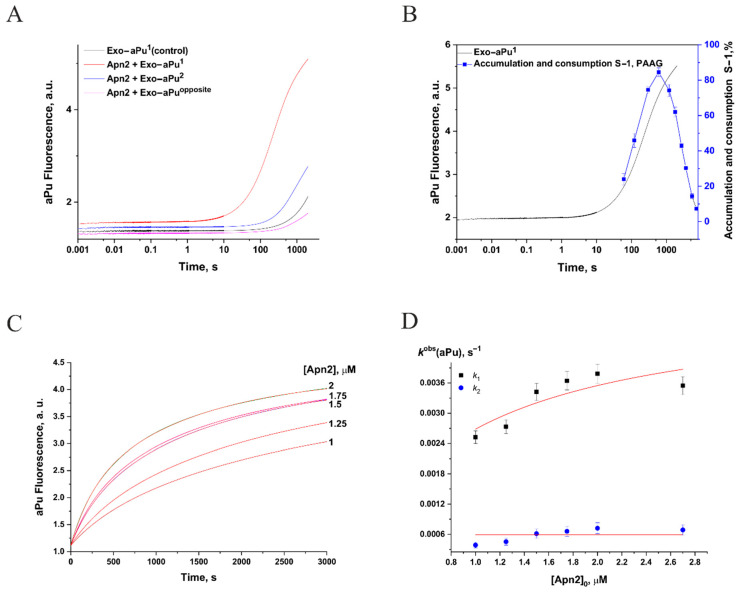
(**A**) Changes in fluorescence intensity of aPu during the interaction of Apn2 with Exo–aPu^1^, Exo–aPu^2^, or Exo–aPu^opposite^ ([S] = 1 μM, [Apn2] = 2 μM, T = 37 °C). (**B**) The comparison between kinetic curves built by means of the change in aPu fluorescence intensity during the interaction of Apn2 with Exo–aPu^1^ and those built by means of the accumulation of reaction products in a PAAG ([S] = 1 μM, [E] = 2 μM, T = 37 °C). (**C**) Changes in fluorescence intensity of aPu during the interaction of Apn2 with Exo–aPu^1^ ([S] = 1 μM, [Apn2] was varied from 1 to 2 μM). (**D**) The dependence of observed rate constants *k*_1_^aPu^ and *k*_2_^aPu^ on Apn2 concentration during its interaction with Exo–aPu^1^.

Figure 7A shows kinetic curves of changes in fluorescence intensity of aPu during the interaction with Apn2. For substrates containing aPu at the first and second positions from the 3′ end of the hydrolyzable strand, growth of the fluorescence intensity of aPu was seen with time, starting from time point 50 s for Exo–aPu^1^ and 200 s for Exo–aPu^2^. For substrate Exo–aPu^opposite^, the change in fluorescence intensity of aPu was comparable to that in the control curve (without the enzyme); accordingly, for this substrate, the interaction with the enzyme probably did not cause a substantial change in the environment of aPu. It should be noted that no changes in aPu fluorescence intensity were detectable at time points up to 10 s, suggesting that the use of this fluorescent analog does not permit the registration of conformational transitions in DNA at the stage of enzyme–substrate complex formation. The increase in fluorescence intensity of aPu for substrate Exo–aPu^1^ at time points > 50 s represents detachment of aPu from the 3′ end of the oligonucleotide as suggested by PAAG electrophoresis experiments (Figure 7B).

For substrate Exo–aPu^1^, kinetic curves of changes in aPu fluorescence intensity were obtained for different concentrations of the enzyme (Figure 7C). It is evident that with an increase in the concentration of the enzyme relative to the substrate from an equimolar ratio to a twofold excess, the slope of the curves changes, indicating that at a higher concentration of the enzyme, the catalytic stage of the 3′–5′–exonuclease reaction proceeds faster. To determine the observed rate constant of the 3′–5′–exonuclease reaction, each kinetic curve was fitted to a two–exponential Equation (2). Observed rate constant *k*_1_^aPu^, corresponding to the phase of the maximal increase in aPu fluorescence intensity, had a hyperbolic dependence on the concentration of Apn2 (Figure 7D). With the help of Equation (1), substrate–binding constant *K*_S_ (1.1 ± 0.5 μM^−1^) and catalytic step rate constant *k*_cat_ (0.005 ± 0.001 s^−1^) were computed. The constants are in good agreement with the data from PAAG electrophoresis (Table 1). Observed rate constant *k*_2_^aPu^, which characterizes the second slow stage, was found to not depend on the Apn2 concentration and probably reflects the process of dissociation of the nucleotide containing aPu from the enzyme active site (Figure 7D).

**Figure 8:**
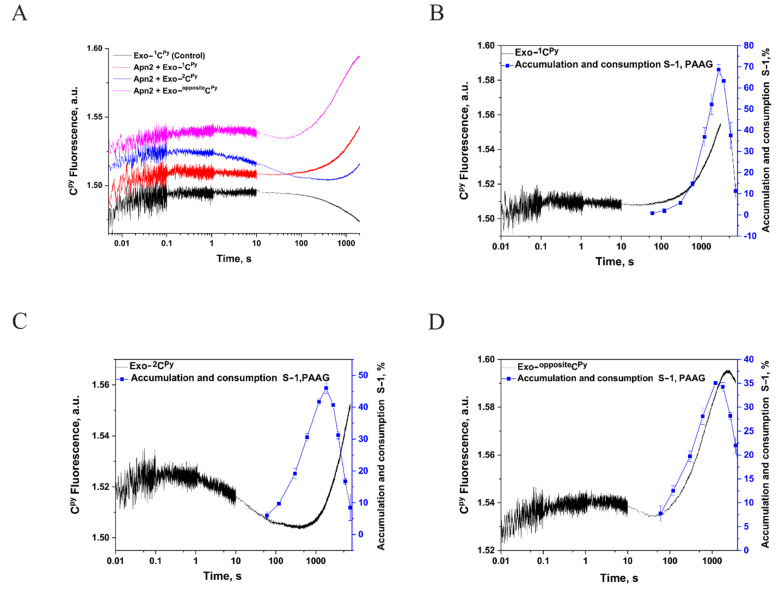
(**A**) Time dependence of the fluorescence intensity of C^py^ during the interaction of Apn2 with DNA duplexes containing this fluorescent label at one of three positions. A comparison of kinetic curves recorded using changes in C^py^ fluorescence intensity during the interaction of Apn2 with DNA duplexes Exo–^1^C^py^ (**B**), Exo–^2^C^py^ (**C**), or Exo–^opposite^C^py^ (**D**) and those recorded using the accumulation of reaction products in a PAAG ([S] = 1 μM, [E] = 2 μM, T = 37 °C).

When C^py^ served as the fluorescent probe, it was possible to register conformational changes in DNA at the stage of the formation of the enzyme–substrate complex. Figure 8A depicts the time dependence of C^py^ fluorescence intensity during the interaction of Apn2 with DNA substrates containing C^py^ at various positions.

In the case of DNA substrate Exo–^1^C^py^, which contains 3′–terminal C^py^, an increase in fluorescence intensity was seen starting with time point 100 s (Figure 8A,B), meaning a shift in the hydrophobicity of the environment of C^py^. This process most likely reflects the cleaving off of the 3′–terminal nucleotide and its release from the active site of the enzyme, consistent with the curve of accumulation of the S–1 product of hydrolysis obtained by PAAG electrophoretic analysis (Figure 8B).

When C^py^ was located at the second position from the 3′ end of the hydrolyzable strand, for the Exo–^2^C^Py^ substrate (Figure 8C), at time points up to approximately 200 s, a slow decline in fluorescence intensity and then its subsequent rise was noted. Probably, this drop matches the stage of enzyme translocation along the DNA duplex after the hydrolytic removal of the first nucleotide. It should also be pointed out that the subsequent rise in the curve probably means for the formation of the S–2 product. This notion is in good agreement with the curve of consumption of the S–1 product because the formation of product S–2 matches the consumption of S–1.

During the interaction of Apn2 with a substrate containing C^py^ in the complementary strand opposite the nucleotide being cleaved off, for substrate Exo–^opposite^C^Py^ (Figure 8D), at time points up to 20 s, there was a decrease in C^py^ fluorescence intensity, which presumably reflects conformational rearrangements of the enzyme–substrate complex and the acquisition of a catalytically competent state by this complex. Further growth of fluorescence, as in the previous case, is suggestive of a change in the hydrophobicity of the C^py^ environment owing to the cleavage of the 3′–terminal nucleotide.

## 3. Materials and Methods

In this work, reagents from Sigma–Aldrich (St. Louis, MO, USA) were used: acrylamide, N,N′–methylene bisacrylamide, 2–amino–2–(hydroxymethyl)–1,3–propanediol (Tris), tetramethylethylenediamine (TEMED), urea, boric acid, dithiothreitol (DTT), ethylenediaminetetraacetic acid (EDTA) and its sodium salt, Coomassie Brilliant Blue R–250, ammonium peroxodisulfate, 4–(2–hydroxyethyl)–1–piperazineethanesulfonic acid (HEPES), sodium dodecyl sulfate (SDS), magnesium chloride, potassium chloride, and glycerol. All solutions were prepared with double–distilled water.

### 3.1. DNA Substrates

Because Apn2 manifests the highest 3′–5′–exonuclease activity on double–stranded DNA substrates having either blunt ends or a 5′–overhang [38], we employed 17–mer duplexes with a 5′ overhang as a simplified model of a substrate containing a single–strand break. Namely, oligonucleotides modified with 2–aminopurine (aPu), pyrrolocytosine (C^py^), or 6–carboxyfluorescein (FAM) (Table 2) as part of a DNA duplex served as DNA substrates.

### 3.2. The Enzyme

The wild–type enzyme Apn2 was isolated from *Escherichia coli* Arctic DE3 cells transformed with the pET28c plasmid carrying the *S. cerevisiae* AP endonuclease gene by the following procedure. *E. coli* Arctic DE3 cells were cultivated at 37 °C in the 2YT medium (1 L) containing 20 µg/mL gentamicin, 10 µg/mL tetracycline, and 50 µg/mL kanamycin to an optical density of 0.6–0.7 at a wavelength of 600 nm. After that, the temperature was lowered to 16 °C, and transcription was induced by the addition of isopropyl–β–d–thiogalactopyranoside to a concentration of 0.2 mM. The cell culture was incubated for 24 h. The cells were then pelleted by centrifugation for 20 min at 5000× *g* at 4 °C, and a suspension of the cells was prepared in 30 mL of a buffer composed of 20 mM HEPES–NaOH pH 7.8, 40 mM NaCl, and a mixture of protease inhibitors (Inhibitor cocktail, Complete, Mannheim, Germany). The resuspended cells were lysed using a French press. All subsequent procedures were carried out at 4 °C. The resultant cell lysate was centrifuged (40 min at 40,000× *g*). Then, a solution of NaCl and imidazole was added to the supernatant to concentrations of 500 and 20 mM, respectively. The resulting solution was mixed with 1 mL of the Ni Sepharose^TM^ High Performance resin (Amersham Biosciences, Uppsala, Sweden) and stirred for 1 h. The enzyme was eluted with 5 mL of a buffer consisting of 20 mM HEPES–NaOH pH 7.8, 500 mM NaCl, and 600 mM imidazole. The obtained enzyme–containing fraction was diluted to a final NaCl concentration of 40 mM and applied to a HiTrap–Heparin™ column (Amersham Biosciences, Uppsala, Sweden) at a flow rate of 0.4 mL/min. The chromatography was performed in a buffer containing 20 mM HEPES–NaOH and a linear 40 → 1000 mM gradient of NaCl; optical density of the solution was recorded at 280 nm. The purity of the Apn2 protein was determined by gel electrophoresis (Figure S1). Fractions containing the Apn2 protein were dialyzed against a buffer (20 mM HEPES–NaOH pH 7.5, 1 mM DTT, 100 mM NaCl, and 20% of glycerol) and stored at −20 °C. The enzyme concentration was calculated from the optical density of the protein at 280 nm and a molar extinction coefficient of 50,475 M^−1^·cm^−1^.

Experiments were conducted in a buffer composed of 50 mM Tris–HCl (pH 7.5), 1 mM EDTA, 3 mM MgCl_2_, 50 mM KCl, 1 mM DTT, and 7% glycerol unless stated otherwise.

### 3.3. Electrophoresis in a Denaturing Polyacrylamide Gel (PAAG)

Analysis of the hydrolysis of model DNA substrates was performed via the following procedure. To 10 μL of a buffer containing a substrate, 10 μL of the enzyme in a buffer was added at 37 °C. The concentration ratio of the enzyme to substrate and time were varied depending on the type of experiment. To obtain kinetic dependences of the degree of substrate cleavage on time, to 50 µL of a buffer containing 2 µM substrate, 50 µL of the enzyme was added at concentrations of 1, 1.6, 2, 3, 4, or 5 µM in the same buffer. The reaction was stopped by adding 20 μL of a solution containing 7 M urea and 25 mM EDTA.

To find the optimal MgCl_2_ concentration, a buffer was utilized in which the Mg^2+^ concentration was varied from 0 to 10 mM. To determine the optimal KCl concentration, a buffer was used in which the KCl concentration was varied from 0 to 300 mM. To find the optimal pH of the solution, a buffer was employed in which pH was varied from 6.5 to 8.5.

To determine the effect of 2′–deoxyribonucleoside monophosphates on the Apn2 enzymatic activity, 10 mM dNMP was added to 40 µL of a buffer containing 2 µM enzyme and incubated for 5 min. Next, a 10 µL aliquot was taken and added to 10 µL of 2 µM substrate in the same buffer; the reaction time was 10 min.

Separation of the products by electrophoresis was carried out in a 15% PAAG under denaturing conditions (7 M urea) in a vertical thermostatted Protean II xi unit (Bio–Rad Laboratories, Inc., Hercules, CA, USA) with voltage 200–300 V at 50 °C. The gel was visualized by means of an E–Box CX.5 TS gel documentation system (Vilber Lourman, Collégien, France).

The degree of substrate transformation was determined in the Gel–Pro Analyzer 4.0 software (Media Cybernetics, Rockville, MD, USA). The degree of transformation was computed as the ratio of the sum of peak areas of cleavage products to the sum of the peak areas of the products and of a starting oligodeoxyribonucleotide. Expected error in determination of the degree of transformation, as a rule, did not exceed 20%.

### 3.4. The Stopped–Flow Method

Kinetic curves of fluorescence intensity were recorded on an SX.20 stopped–flow spectrometer (Applied Photophysics, Leatherhead, UK). The instrument’s dead time is 1.0 ms. As fluorophores, aPu and C^py^ were used, as was amino acid residue tryptophan contained in the protein. Registration of the data was conducted under conditions close to one turnover of the enzyme, i.e., at enzyme and substrate concentrations of the same order of magnitude. These experiments were conducted essentially as described previously [38,39,40,41].

The enzyme Apn2 (500 µL) and duplex DNA (500 µL) in a buffer at various concentrations were placed into syringes, then the solutions were rapidly mixed. The reaction was carried out at 37 °C.

To calculate rate constants of conformational transitions, we employed the OriginLab 15.0 software (OriginLab Corp., Northampton, MA, USA), which helped to compute parameters of the catalytic process (kinetic constants) by the nonlinear regression method.

To calculate the observed rate constants characterizing a change in fluorescence intensity, namely, *k_i_*^obs^, the kinetic curves were fitted to the equation

(2) $$F(t)=F_{0}+F_{1}\times (1-e^{-k1^{\mathrm{obs}}{}^{\times t}})+F_{2}\times (1-e^{-k2^{\mathrm{obs}}{}^{\times t}})$$

where *F*(*t*) is observed fluorescence intensity at time *t*, *F*_0_ denotes a background signal level, *F*_1_ means the maximum amplitude of fluorescence intensity change at *t* → ∞, and *k*^obs^ is the observed rate constant. Under quasi–equilibrium conditions, *k*^obs^ is expressed as

*k*^obs^ = *k*_1_[S] + *k*_−1_ (3)

where [S] represents substrate concentration, and *k*_1_ and *k*_−1_ are rate constants for the formation and dissociation of the enzyme–substrate complex.

## 4. Conclusions

In this study, pre–steady–state kinetics of conformational transformations of the enzyme Apn2 and model DNA substrates were investigated by the stopped–flow method with registration of changes in fluorescence intensity of this protein’s tryptophan residues as well as in the fluorescence of aPu or C^py^ residues in DNA. It is shown for the first time that Apn2 and DNA undergo conformational transformations during their interaction. According to these experimental data, a kinetic scheme was derived that describes the mechanism behind the interaction of Apn2 with DNA substrates, and we determined the constant for the enzyme–substrate complex formation and rate constants of the reactions included in the kinetic scheme.

During the interaction of Apn2 with a DNA substrate, the formation of an enzyme–substrate complex (E•DNA_n_) takes place at the first stage; this process was registered by means of the changing fluorescence of Trp and C^py^ in substrates Exo–^opposite^C^Py^ and Exo–^2^C^Py^. The change of fluorescence intensity in substrate Exo–^opposite^C^Py^ between 10 s and 100 s indicates the process of conformational rearrangements, probably induced by the formation of a catalytically competent enzyme–substrate complex. By means of changes in fluorescence intensity of C^py^ and aPu located at the 3′ end of the hydrolyzable strand (substrates Exo–^1^C^Py^ and Exo–aPu^1^) at time points > 100 s, the catalytic stage of the process was detected. For the substrate containing C^py^ at the second position of the 3′ terminus of the hydrolyzable strand (Exo–^2^C^Py^), an additional stage of fluorescence intensity alternation was documented, most likely explained by the translocation of the enzyme and by the assembly of a new catalytically competent enzyme–substrate complex.

An influence of the nature of the cleaved off 3′–terminal nucleotide on the course of the 3′–5′–exonuclease reaction catalyzed by Apn2 is demonstrated. It is shown that the efficiency of the enzymatic reaction is context–specific, while the preferred nucleotide to be removed is 2′–deoxyadenosine, whereas the presence of a noncomplementary pair at the 3′ end raises the rate of degradation of the substrate. It should be noted that the Apn2–mediated mechanism of DNA degradation at single–strand breaks can be associated with the action of Apn1 and probably other BER proteins such as PCNA, as demonstrated for human APE1/APE2 [31]. Therefore, it can be hypothesized that these proteins can influence the 3′–5′–exonuclease activity of Apn2 and affect substrate specificity of the enzyme toward the DNA context. Otherwise, the obtained data indicate that the release of 2′–deoxyribonucleoside monophosphates from the active site of the enzyme is not a limiting factor for the 3′–5′–exonuclease reaction, as previously documented for human APE1 [22] and *E. coli* Nfo [23]. It can be proposed that the biological function of the Apn2 enzyme in sequential two–stage endo–exonucleolytic degradation of DNA intermediates generated by the BER pathway is related to this difference in the molecular mechanisms between the aforementioned enzymes.

Thus, this study on pre–steady–state kinetics of tryptophan residues’ fluorescence in the enzyme and of aPu and C^py^ fluorescence in DNA made it possible to register conformational changes in Apn2 and its DNA substrates during the 3′–5′–exonuclease reaction and to assign them to certain stages of the enzymatic process.

## Figures and Tables

**Table 1:** *K*_s_ and *k*_cat_ values corresponding to the interaction of Apn2 with DNA duplexes.

DNA Substrate	*K*_s_, μM^−1^	*k*_cat_, ×10^3^ s^−1^
^FAM^Exo–C/G	0.40 ± 0.20	2.7 ± 1.2
^FAM^Exo–G/C		3.0 ± 0.3
^FAM^Exo–T/A		3.3 ± 1.2
^FAM^Exo–A/T		7.5 ± 0.7
^FAM^Exo–C/A		13.3 ± 3.3
^FAM^Exo–C/T		12.0 ± 0.2
^FAM^Exo–C/C		16.7 ± 5.0
^FAM^Exo–CCC/GGG	1.0 ± 1.2	2.4 ± 0.4
^FAM^Exo–GGG/CCC	0.3 ± 0.4	3.4 ± 1.6
^FAM^Exo–TTT/AAA	0.7 ± 0.4	2.2 ± 0.1
^FAM^Exo–AAA/TTT	7.2 ± 4.1	62 ± 25

**Table 2:** DNA duplexes used as substrates.

Name	Sequence
^FAM^Exo–X/Y, where X=G/A/T/C, Y=G/A/T/C	5′–FAM–CAGCTCTGTACGTGAG**X–**3′ 3′–GTCGAGACATGCACTC**Y**TCACCACTGTG–5′
^Trp^Exo–G/C	5′–CAGCTCTGTACGTGAG**G–**3′ 3′–GTCGAGACATGCACTC**C**TCACCACTGTG–5′
^FAM^Exo–CCC/GGG	5′–FAM–CAGCTCTGTACGTG**CCC–**3′ 3′–GTCGAGACATGCAC**GGG**TCACCACTGTG–5′
^FAM^Exo–GGG/CCC	5′–FAM–CAGCTCTGTACGTG**GGG–**3′ 3′–GTCGAGACATGCAC**CCC**TCACCACTGTG–5′
^FAM^Exo–TTT/AAA	5′–FAM–CAGCTCTGTACGTG**TTT–**3′ 3′–GTCGAGACATGCAC**AAA**TCACCACTGTG–5′
^FAM^Exo–AAA/TTT	5′–FAM–CAGCTCTGTACGTG**AAA–**3′ 3′–GTCGAGACATGCAC**TTT**TCACCACTGTG–5′
Exo–aPu^1^	5′–CAGCTCTGTACGTGAG**aPu–**3′ 3′–GTCGAGACATGCACTC **T** TCACCACTGTG–5′
Exo–aPu^2^	5′–CAGCTCTGTACGTGA**aPu**T**–**3′ 3′–GTCGAGACATGCACT **T** ATCACCACTGTG–5′
Exo–aPu^opposite^	5′–CAGCTCTGTACGTGAG **T–**3′ 3′–GTCGAGACATGCACTC**aPu**TCACCACTGTG–5′
Exo–^1^C^Py^	5′–CAGCTCTGTACGTGAG**C^py^–**3′ 3′–GTCGAGACATGCACTC**G**TCACCACTGTG–5′
Exo–^2^C^Py^	5′–CAGCTCTGTACGTGA**C^py^**T**–**3′ 3′–GTCGAGACATGCACT **G** ATCACCACTGTG–5′
Exo–^opposite^C^Py^	5′–CAGCTCTGTACGTGAG**G–**3′ 3′–GTCGAGACATGCACTC**C^py^**TCACCACTGTG–5′

## Data Availability

Raw experimental data are available from N.A.K. upon request. Tel.: +7-(383)-363-5174, E-mail: nikita.kuznetsov@niboch.nsc.ru.

## Supplementary Materials

The following supporting information can be downloaded at: https://www.mdpi.com/article/10.3390/ijms232214404/s1.

## Abbreviations

AP site: apurinic/apyrimidinic site; Apn2: AP endonuclease 2 from *Saccharomyces cerevisiae*; aPu: 2–aminopurine; BER: base excision repair; C^py^: pyrrolocytosine; dNMP: 2′–deoxynucleoside monophosphate; FAM: 6–carboxyfluorescein; MST: microscale thermophoresis; NIR: nucleotide incision repair; PAAG: polyacrylamide gel.
