# Supplementary material for: Kinetic Features of 3′–5′–Exonuclease Activity of Apurinic/Apyrimidinic Endonuclease Apn2 from Saccharomyces cerevisiae
Source: Int J Mol Sci. 2022 Nov 19;23(22):14404. doi: 10.3390/ijms232214404 (PMC9697762; doi:10.3390/ijms232214404)
Supplement: Supplementary file 1 [file ijms-23-14404-s001.zip › ijms-1988372-supplementary.pdf]

Supplementary material

Kinetic Features of 3′–5′–Exonuclease Activity of Apurinic/Apyrimidinic Endonuclease Apn2 from *Saccharomyces cerevisiae*

Aleksandra A. Kuznetsova [1], Anastasia A. Gavrilova [1], Alexander A. Ishchenko [2], Murat Saparbaev [2], Olga S. Fedorova [1,†] and Nikita A. Kuznetsov [1,3,*]

[1] Institute of Chemical Biology and Fundamental Medicine, Siberian Branch of Russian Academy of Sciences, 630090 Novosibirsk, Russia

[2] Group «Mechanisms of DNA Repair and Carcinogenesis», Equipe Labellisée LIGUE 2016, CNRS UMR9019, Université Paris–Saclay, Gustave Roussy Cancer Campus, CEDEX, F-94805 Villejuif, France

[3] Department of Natural Sciences, Novosibirsk State University, 630090 Novosibirsk, Russia

* Correspondence: nikita.kuznetsov@niboch.nsc.ru

† deceased.

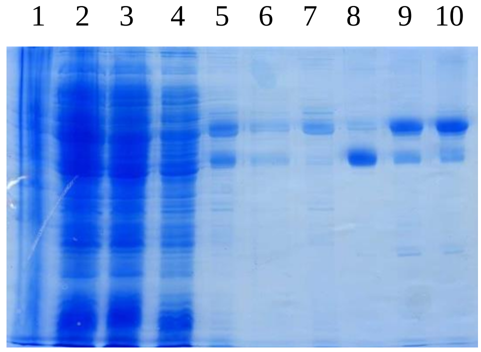

Figure S1. The 12% SDS-PAAG of Apn2 purification procedure. Lanes: 1 - precipitate after cells lysis, 2 - lysate after cells lysis, 3-4 – fractions unbound with Ni-Sepharose™ sorbent, 5-6 - fractions bound with Ni-Sepharose™ sorbent, 7 – fraction unbound with HiTrap-Heparin™ column, 8-9 - Apn2 fractions obtained during chromatography in a linear gradient of 40 → 1000 mM NaCl, 10 - control (Apn2).

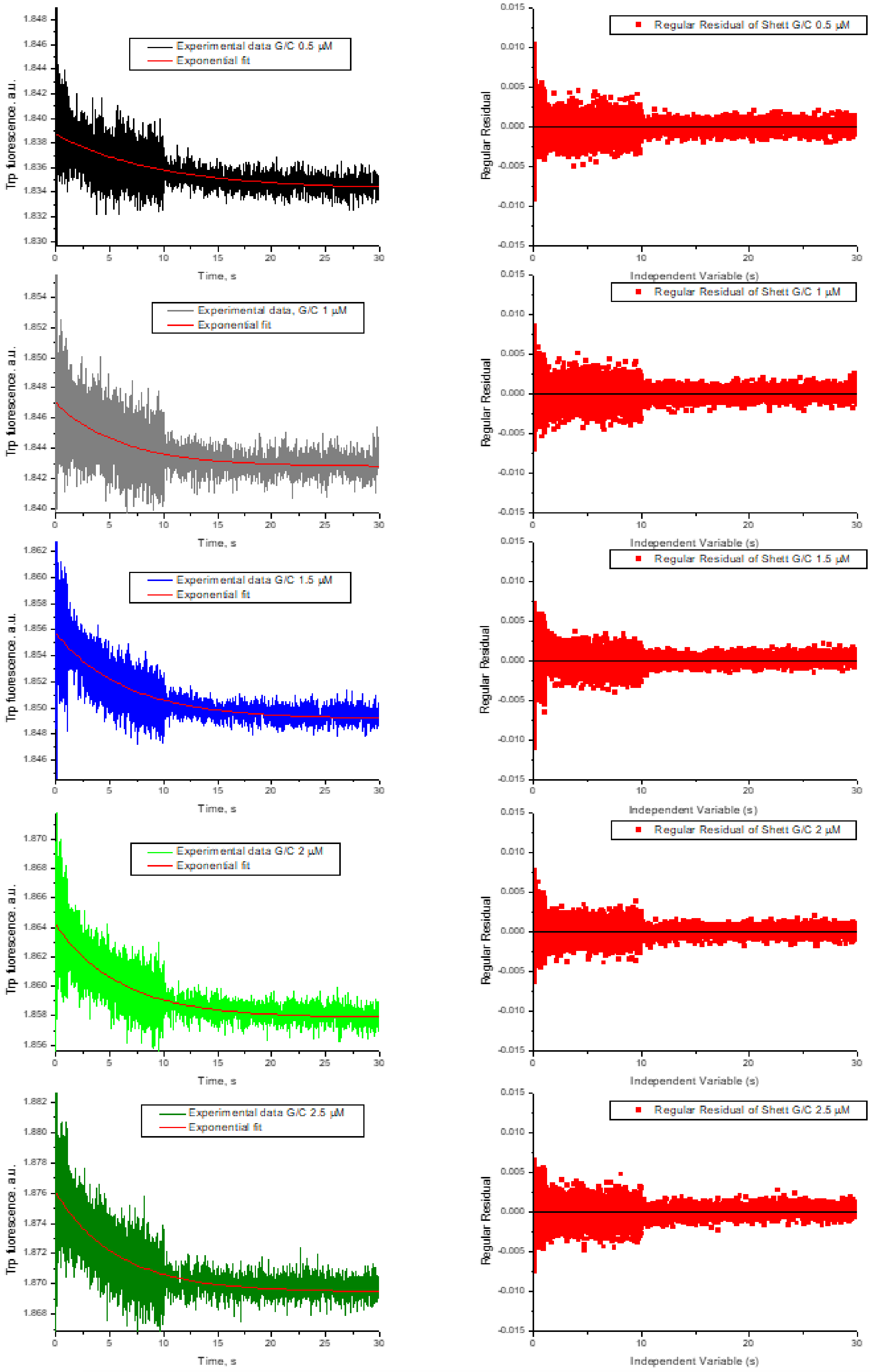

Figure S2. The experimental and theoretical kinetic curves (left panels) and regular residuals of fit curves (right panels), obtained for interaction of Apn2 with ^{Trp}Exo-G/C substrate.
